# Cytotoxic effects of ZnO hierarchical architectures on RSC96 Schwann cells

**DOI:** 10.1186/1556-276X-7-439

**Published:** 2012-08-08

**Authors:** Yixia Yin, Qiang Lin, Haiming Sun, Dan Chen, Qingzhi Wu, Xiaohui Chen, Shipu Li

**Affiliations:** 1 State Key Laboratory of Advanced Technology for Materials Synthesis and Processing, and Biomedical Materials and Engineering Center, Wuhan University of Technology, Wuhan, 430070, People’s Republic of China; 2 Department of Prosthetic, School of Stomatology, Wuhan University, Wuhan, 430079, People’s Republic of China

**Keywords:** ZnO, Hierarchical architectures, Neurotoxic effect

## Abstract

The alteration in intracellular Zn^2+^ homeostasis is attributed to the generation of intracellular reactive oxygen species, which subsequently results in oxidative damage of organelles and cell apoptosis. In this work, the neurotoxic effects of ZnO hierarchical architectures (nanoparticles and microspheres, the prism-like and flower-like structures) were evaluated through the 3-(4, 5-dimethylthiazol-2-yl)-2, 5-diphenyltetrazolium bromide assay using RSC96 Schwann cells as the model. Cell apoptosis and cell cycle were detected using flow cytometry. The concentration of Zn^2+^ in the culture media was monitored using atomic absorption spectrometry. The results show that ZnO nanoparticles and microspheres displayed significant cytotoxic effects on RSC96 Schwann cells in dose- and time-dependent manners, whereas no or low cytotoxic effect was observed when the cells were treated with the prism-like and flower-like ZnO. A remarkable cell apoptosis and G2/M cell cycle arrest were observed when RSC96 Schwann cells were exposed to ZnO nanoparticles and microspheres at a dose of 80 μg/mL for 12 h. The time-dependent increase of Zn^2+^ concentration in the culture media suggests that the cytotoxic effects were associated with the decomposition of ZnO hierarchical architecture and the subsequent release of Zn^2+^. These results provide new insights into the cytotoxic effects of complex ZnO architectures, which could be prominently dominated by nanoscale building blocks.

## Background

ZnO nanostructures have attracted global interest because of their excellent optoelectronic, piezoelectric, ferromagnetic, and optical properties. Therefore, evaluating the biocompatibility of ZnO nanostructures is important. However, contradictory results on the biocompatibility of ZnO nanostructures were reported in numerous studies [1-6]. For example, no or low cytotoxic effect was observed on rat L2 lung epithelial cells and primary rat lung alveolar macrophage treated with ZnO nanoparticles (NPs) [2]. ZnO nanowires were shown to be completely biocompatible on HeLa cells but cytotoxic on L929 cells at a dose of 100 μg/mL [3]. *In vitro* experiments indicated that ZnO NPs have potential applications in cancer diagnosis and therapy [7-9]. However, significant cytotoxic effects were observed when cells were treated with ZnO nanostructures [10-16]. For example, the treatment of different cells (such as epithelial A549, A431, BEAS-2B cells, and macrophage RAW 264.7 cells) with ZnO NPs induces remarkable intracellular oxidative stress and DNA damage [17-19]. To date, only a few investigations have evaluated the cytotoxicity of complex ZnO nanostructures assembled by nanoscale building blocks.

Zn^2+^ is a vital component of enzymes and proteins and an ionic signal among various intracellular organelles and storage depots [20]. It modulates protein function by binding to and detaching from intracellular zinc-dependent proteins. Nevertheless, excess free Zn^2+^ is cytotoxic and can induce serious neuronal injury [21-23]. Considerable evidence shows that free Zn^2+^ in the extracellular fluid results in amyloid deposition, one of the pathological hallmarks of Alzheimer’s disease [24,25]. To date, little is known about the neurotoxic effects of ZnO nanostructures. In the present study, the neurotoxic effects of ZnO hierarchical architectures (including NPs and hollow microspheres consisting of NPs, the prism-like and flower-like structures) were evaluated using RSC96 Schwann cells as the model. RSC96 Schwann cells are the main supportive cells of the peripheral nervous system and are responsible for the myelination of axons. Cell viability was measured through the 3-(4, 5-dimethylthiazol-2-yl)-2, 5-diphenyltetrazolium bromide (MTT) assay, and flow cytometry was employed to analyze cell apoptosis and cell cycle. The decomposition of ZnO hierarchical architectures in cell culture media was measured using atomic absorption spectrometry.

## Methods

### Synthesis of ZnO hierarchical architectures

ZnO with different morphologies (the prism-like, flower-like structures, and hollow microspheres) was synthesized according to the method reported previously [26]. Briefly, Zn(CH_3_COO)_2_·2H_2_O (0.2195 g, 1 mmol) was dissolved in 25 mL deionized water in the magnetic stirring, and then histidine (His, 0.1552 g, 1 mmol) was added into the zinc acetate solution. NaOH (0.88 g, 22 mmol) was dissolved in 15 mL deionized water and added dropwise into the solution containing zinc acetate and His. After 15 min stirring, the mixture was transferred to and sealed in a 50-mL Teflon-lined autoclave, heated to 150°C for 10 h, then finally cooled to room temperature. In the series of the synthesis, the amount of NaOH and His was changed at the designed molar ratios. The precipitate was collected by the centrifugation (10,000 rpm, 5 min), washed alternately with the deionized water and ethanol, and dried in air at 60°C for 4 h. In order to prepare ZnO NPs, Zn(CH_3_COO)_2_·2H_2_O (0.2195 g, 1 mmol) was dissolved in 37 mL EG. NaOH (0.1 g, 2.5 mmol) was dissolved in 3 mL deionized water and added to Zn(CH_3_COO)_2_ solution under magnetic stirring. The mixture was transferred to and sealed in a 50-mL Teflon-lined autoclave and heated to 150°C for 10 h.

### Characterization

The morphology and structure of ZnO hierarchical architectures obtained were observed through field-emission scanning electron microscopy (FESEM, Sirion 200, FEI Corp., Eindhoven, Netherlands) and transmission electron microscopy (TEM, Tecnai G2-20, FEI Corp., Eindhoven, Netherlands), respectively.

### MTT assay of cell viability

ZnO hierarchical architectures were ultrasonically dispersed in phosphate buffer solution (PBS) and added to cell culture media at the designed doses. The cell viability was measured using the MTT assay. Briefly, RSC96 Schwann cells were seeded in a 96-well plate at a density of 1 × 10^5^ cells/mL. The cells grew for 12 h after seeding and were treated with ZnO hierarchical architectures at designed doses (4, 8, 40, 80, and 400 μg/mL) for different times (6, 12, 24, and 48 h). A 20-μL MTT (5 mg/mL) was added to each well and incubated for 4 h after removing zinc compounds-containing culture media and washing the cells with PBS three times. Finally, all media were removed and 150 μL DMSO was added to each well and shaken for 10 min. The absorbance was read at a wavelength of 550 nm using a Benchmark Microplate Reader (Bio-Rad Corp., Hercules, CA, USA).

### Apoptosis and cell cycle analysis

To assay the percentage of apoptotic and necrotic cells, FITC-annexin V- and propidium iodide (PI)-stained cells were analyzed using an Annexin V-FITC detection kit (BD Pharmingen Inc., San Diego, CA, USA) according to the manufacturer’s instructions. RSC96 Schwann cells were seeded in a 12-well plate at a density of 1 × 10^5^ cells/mL. The cells were allowed to grow for 12 h after seeding and were treated with ZnO hierarchical architectures at doses of 8 and 80 μg/mL for 12 h, respectively. After being washed thrice with ice-cold PBS, the cells were resuspended in 400 μL binding buffer (10 mM HEPES/NaOH, pH 7.4, 150 mM NaCl, 5 mM KCl, 1 mM MgCl_2_, and 1.8 mM CaCl_2_) at a density of 8 × 10^6^ cells/mL. Subsequently, they were filtered with a 100-μm filter and then co-incubated with 5 μL FITC-annexin V (25 μg/mL) and 1 μL PI (50 μg/mL) in the absence of light for 15 min at room temperature. Finally, the fluorescence intensities of the stained cells were analyzed using a FACScalibur Flow cytometer (Becton, Dickinson and Company, Franklin Lakes, NJ, USA).

To assay the cell cycles, the cells were resuspended in ice-cold 70% ethanol and then incubated at 4°C for 1 h. The samples were stored at −20°C for 24 h. After being centrifuged at 150 × *g* for 8 min, the cells were washed twice with ice-cold PBS and then co-incubated with RNase (60 μg/mL) at 37°C for 30 min. The mixture was cooled in an ice bath for 2 min to stop the digestion of RNase. Then, 500 μL PI (50 μg/mL) was added and incubated in the absence of light for at least 30 min at 4°C. After being filtered with a 100-μm filter, the samples were transferred and analyzed using a flow cytometer. Cell cycle was assessed using a FACScan flow cytometer (BD Biosciences, San Jose, CA, USA), CellQuest software (version 2.0) and ModFit LT (Verity Software House, version 2.0, Topsham, ME, USA). The percentage of cells in sub-Go/G1, Go/S, and G2/M phases was analyzed using ModFit LT (Verity Software House, version 2.0).

### Measurement of Zn^2+^ concentration in culture media

Culture media were collected and centrifuged (10,000 rpm, 5 min) after cells were incubated with ZnO hierarchical architectures at designed times and doses. The suspension was carefully collected for the measurement of Zn^2+^ concentration using atomic absorption spectrometry.

## Results and discussion

**Figure 1:**
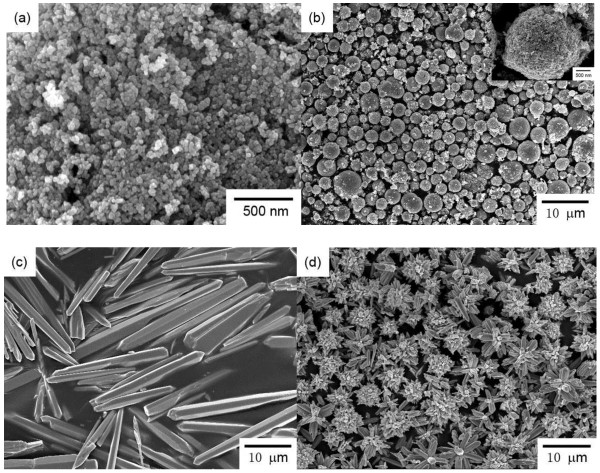
**SEM images of the ZnO hierarchical architectures used for the cytotoxic assessment on RSC96 Schwann cells.** (**a**) ZnO NPs, (**b**) hollow ZnO microspheres, (**c**) prism-like ZnO, and (**d**) flower-like ZnO.

**Figure 2:**
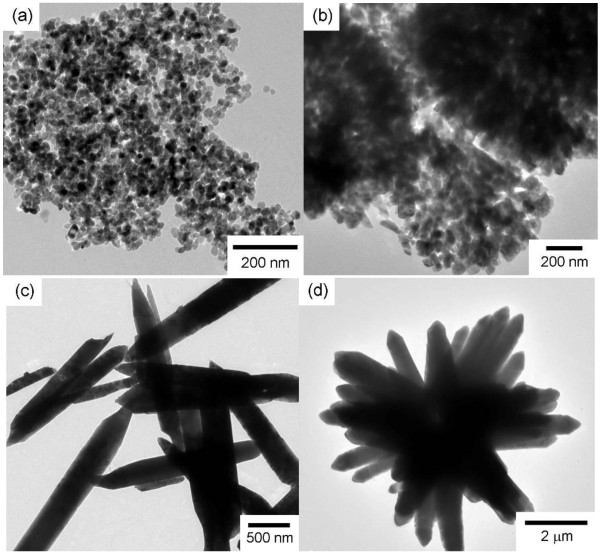
**TEM images of the ZnO hierarchical architectures used for the cytotoxic assessment on RSC96 Schwann cells.** (**a**) ZnO NPs, (**b**) hollow ZnO microspheres, and (**c**) prism-like ZnO, and (**d**) flower-like ZnO.

The morphologies of ZnO hierarchical architectures were characterized through scanning electron microscopy (SEM) and TEM observation. As shown in Figures 1a and 2a, monodispersed spherical ZnO NPs with an average diameter of ca. 35 nm were obtained. Hollow ZnO microspheres with an average diameter of ca. 2.7 μm were also obtained (Figure 1b). Numerous ZnO NPs were adsorbed on the rough surface of the microspheres. As shown in the inset of Figure 1b, the ZnO microspheres consisted of numerous NPs that are slightly larger than those in Figure 1a. The TEM image in Figure 2b further confirms the hollow structure of the microspheres consisting of NPs that are ca. 45 nm in diameter (Figure 2b). A hexahedral, prism-like ZnO was synthesized with a sharp but irregular tip (Figures 1c and 2c). The prism-like structure was ca. 2.5 to 6.0 μm in diameter and ca. 18.0 to 60.0 μm in length. Figures 1d and 2d show SEM and TEM images of the flower-like structure which consisted of sword-like leaves and prism-like pistils. Both the leaves and the prism-like pistils were ca. 500 to 600 nm in diameter (or in width) and several microns in length.

**Figure 3:**
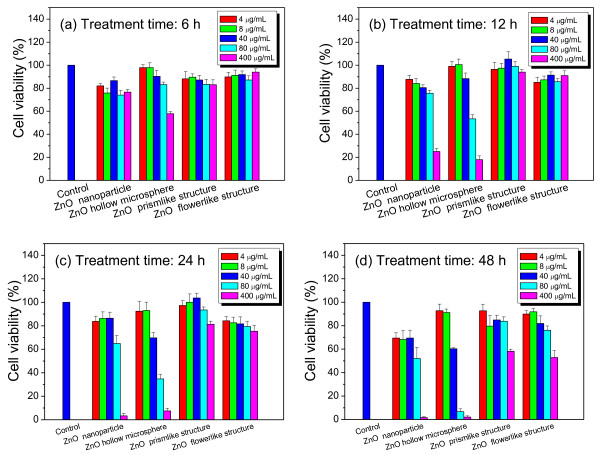
**Effects of ZnO hierarchical architectures on cell viability of RSC96 Schwann cells (*n*** **= 5).** (**a**) Treatment time for 6 h, (**b**) treatment time for 12 h, (**c**) treatment time for 24 h, (**d**) treatment time for 48 h.

The cytotoxicity of the ZnO hierarchical architectures on RSC96 Schwann cells was evaluated via MTT assay. As shown in Figure 3a,b, no or very low cytotoxic effect was observed when RSC96 Schwann cells were treated with the prism-like and flower-like ZnO for 12 h at different doses (4, 8, 40, 80, and 400 μg/mL). Significant cytotoxic effects were observed when the treatment was prolonged up to 48 h at the high dose of 400 μg/mL. Cell viability sharply decreased to ca. 58.2% and 52.8% compared with that of the control. Dose- and time-dependent cytotoxic effects were observed when the cells were treated with ZnO NPs and microspheres. For example, a slight cytotoxic effect was observed when the cells were treated with ZnO NPs for 24 h at low doses (lower than 40 μg/mL). Cell viability was decreased to ca. 86.5% at an exposed dose of 40 μg/mL compared with that of the control. Acute cytotoxic effects were detected when the dose of the ZnO NPs was increased to 400 μg/mL, resulting in a sharp decrease in cell viability to ca. 25% after 12 h treatment. Almost complete death of cells occurred after 24 h of treatment. Remarkable time-dependent cytotoxic effects were observed at high doses (higher than 80 μg/mL). After being treated with ZnO NPs for 6 to 48 h, the cell viability was decreased from ca. 74.1% to 52.0% and from ca. 76.7% to 1.7% at doses of 80 and 400 μg/mL, respectively.

It is noteworthy that both dose- and time-dependent cytotoxic effects were more significant when RSC96 Schwann cells were treated with ZnO microspheres. For example, no cytotoxic effect was observed when the cells were treated at low doses (lower than 8 μg/mL) for 48 h. The significant cytotoxic effects were observed when the dose was higher than 80 μg/mL. For example, cell viability was decreased to ca. 57.8% when RSC96 Schwann cells were treated with ZnO microspheres at a dose of 400 μg/mL for 6 h. Time-dependent cytotoxic effects were observed when the cells were treated at high doses (higher than 40 μg/mL) for 48 h. For example, cell viability decreased to ca. 83.2%, 53.5%, 34.8%, and 6.8% when the cells were treated at a dose of 80 μg/mL for 6, 12, 24, and 48 h, respectively.

**Figure 4:**
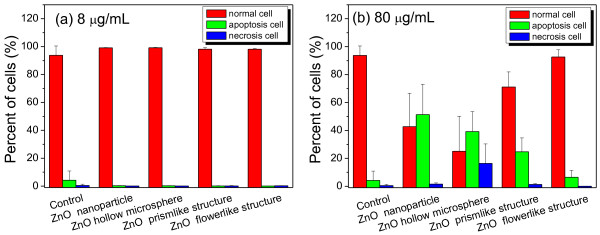
**Flow cytometer analysis of RSC96 Schwann cells treated with ZnO hierarchical architectures for 12 h.** (**a**) at an exposed dose of 8 μg/mL and (**b**) at an exposed dose of 80 μg/mL.

To investigate the cytotoxic effects of ZnO hierarchical architectures on RSC96 Schwann cells, cell apoptosis and necrosis were measured through a flow cytometry. As shown in Figure 4, RSC96 cells treated with ZnO hierarchical architectures at a dose of 8 μg/mL for 12 h grew normally and did not display apoptosis or necrosis, suggesting the absence of significant cytotoxic effects. When the dose was increased to 80 μg/mL, a low apoptosis rate of ca. 24.7% and 6.4% was observed corresponding to the treatment with the prism-like and flower-like ZnO, respectively. By contrast, a significant apoptosis rate of ca. 51.3% and 39.2% was detected when the cells were treated with ZnO NPs and microspheres at a dose of 80 μg/mL for 12 h, respectively. Furthermore, a significant necrosis rate of ca. 16.4% was observed in the cells treated with ZnO microspheres.

The cell cycle distribution was further analyzed to evaluate the cytotoxic effects of ZnO hierarchical architectures. Most cells are usually in the growth phase (G1), wherein various enzymes (especially those needed for DNA replication) are synthesized. In the subsequent S phase, DNA synthesis is completed to double the chromosomes for division. The G2 phase starts from the end of DNA synthesis until the beginning of mitosis, involving the synthesis of microtubules. The M phase lasts throughout the procedure during which the mother cell is divided into two separate but identical daughter cells [27]. In the present study, the major cell population was found in the G1 and S phases in the control group. However, a significant increase of cell population in G2/M phase, accompanied by a decrease of cell population in the G1 and S phases, was observed when RSC96 Schwann cells were treated with ZnO hierarchical architectures at a dose of 8 μg/mL for 12 h (Table 1). These results suggest that the slight G2/M cell cycle arrest was induced by the low-dose treatment of ZnO hierarchical architectures, although no significant cytotoxic effect was detected according to the MTT assay. The population of cells in the G2/M phase was further increased when the cells were treated with ZnO hierarchical architectures at a dose of 80 μg/mL (Table 1), suggesting a more serious G2/M cell cycle arrest than that at the lower dose. These results show that cell cycle was significantly influenced by the treatment of ZnO hierarchical architectures because of serious G2/M cell cycle arrest.

**Table 1:** Cell cycle analysis of RSC 96 Schwann cells after treatment of ZnO hierarchical architectures for 12 h

**Group**	**G1 (%)**		**G2/M (%)**		**S (%)**	
	**8 μg/mL**	**80 μg/mL**	**8 μg/mL**	**80 μg/mL**	**8 μg/mL**	**80 μg/mL**
Control	47.25 ± 0.07		13.55 ± 0.07		39.2 ± 0.14	
ZnO NPs	46.4 ± 1.43	44.5 ± 0.37	19.45 ± 2.18	21.82 ± 0.95	34.15 ± 2.60	33.6 ± 1.27
ZnO microspheres	45.78 ± 1.47	38.68 ± 1.75	16.88 ± 3.15	22.7 ± 3.09	37.35 ± 4.50	38.65 ± 1.80
Prism-like ZnO	47.53 ± 1.00	42.75 ± 0.75	18.43 ± 1.23	21.35 ± 1.88	34.03 ± 1.37	35.85 ± 1.32
Flower-like ZnO	46.05 ± 0.48	40.88 ± 1.32	18.5 ± 0.59	22.55 ± 1.86	35.45 ± 0.39	36.55 ± 1.79

Data were expressed as Mean ± SD (*n* = 5).

**Figure 5:**
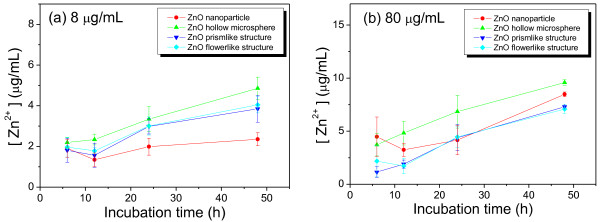
**Changes of Zn**^**2+**^**concentration in the culture medium at different treatment times of ZnO architectures.** (**a**) at tested dose of 8 μg/mL and (**b**) at tested dose of 80 μg/mL.

The decomposition of ZnO hierarchical architectures was measured by monitoring the change of Zn^2+^ concentration in the culture media at different time intervals via atomic absorption spectroscopy. As shown in Figure 5, a significant enhancement of Zn^2+^ concentration was observed with increasing incubation time. This result suggests that the decomposition process occurred during the incubation period. It is noteworthy that the release of Zn^2+^ in the ZnO NPs-treated group was lower than that in the other groups at a dose of 8 μg/mL, but higher than in the prism-like and flower-like ZnO-treated group at a dose of 80 μg/mL. The highest Zn^2+^ concentration was observed in the ZnO microspheres-treated group at both tested doses (e.g., ca. 3.7 to 9.6 μg/mL at time intervals from 6 to 48 h at a dose of 80 μg/mL). These results suggest that at a dose of 80 μg/mL, the release of Zn^2+^ from both ZnO NPs and microspheres was faster than from the flower-like and prism-like ZnO. This result is consistent with the cytotoxic effect observed via the MTT assay.

Compared with bulk counterparts, more atoms are located on the surfaces of smaller NPs, which can interact with biological systems more effectively [28]. In the present work, ZnO NPs and hollow microspheres consisting of NPs displayed more significant cytotoxic effects in dose- and time-dependent manners on RSC96 Schwann cells than the bulk prism-like and flower-like structures. This implies that the cytotoxic effects of complex architectures are most likely predominated by the nanoscale building blocks. The precise cytotoxic mechanisms of ZnO nanostructures still remain indistinct. ZnO NPs can induce a significant accumulation of intracellular reactive oxygen species (ROS) in various cells (such as monocytes and lymphocytes and WIL2-NS human lymphoblastoid cells) in a size-dependent manner. This treatment of ZnO NPs results in the direct alteration of mitochondrial functionality, increase of intracellular Ca^2+^ level, and expression of genes involved in apoptosis and oxidative stress responses [29-31]. In the present work, ZnO NPs and microspheres induced significant cell apoptosis in a dose-dependent manner. The treatment of RSC96 cells with ZnO hierarchical architectures resulted in a remarkable G2/M cell cycle arrest, even at a sublethal dose (8 μg/mL), implying the early DNA damage. Furthermore, the release of Zn^2+^ in the cell culture media was consistent with the cytotoxic effect of ZnO hierarchical architectures on RSC96 Schwann cells. Alterations in Zn^2+^ homeostasis displayed powerful stimulatory effects on multiconductance cation channels in the inner mitochondrial membrane and ROS generation. A loss of Zn^2+^ homeostasis may result in cell apoptosis or necrosis [16,32]. Moreover, the effects of Zn^2+^ level on Ca^2+^ homeostasis were also reported which is another crucial signal pathway closely related to cell apoptosis and necrosis [31,33]. The effects of ZnO NPs on the cell cycle have rarely been studied. Wang et al. [34] reported that the exposure of human embryonic kidney HEK293 cells to SiO_2_ NPs results in the accumulation of cells in the G2/M phase in a dose-dependent manner. AshaRani et al. [35] found that Ag NPs caused a concentration-dependent increase of cell population in the G2/M phase in both normal human lung fibroblast IMR90 cells and human glioblastoma U251cells.

## Conclusions

In summary, the cytotoxic effects of ZnO hierarchical architectures, such as NPs and hollow microspheres consisting of NPs, the prism-like and flower-like structures, were evaluated using RSC96 Schwann cells as the model. The ZnO NPs and microspheres displayed significant cytotoxic effects on RSC96 Schwann cells in time- and concentration-dependent manners. The treatment of cells with ZnO NPs and microspheres induced remarkable cell apoptosis and G2/M cell cycle arrest which were associated with the decomposition of ZnO hierarchical architectures and the subsequent release of Zn^2+^ in the culture media. These results provide new insights into the cytotoxic effects of complex architectures that could be prominently dominated by nanoscale building blocks.

## Competing interests

The authors declare that they have no competing interests.

## Authors’ contributions

YY and XC carried out MTT assay and cell cycle and apoptosis measurement, as well as data analysis. QL, HS, and DC were responsible for the synthesis and characterization of ZnO hierarchical architectures, as well as the measurement of Zn^2+^ concentration in culture media. QW designed the whole work and wrote the manuscript. All authors read and approved the final manuscript.
